# Supplementary material for: Plant Growth-Promoting Bacterial Consortia as a Strategy to Alleviate Drought Stress in Spinacia oleracea
Source: Microorganisms. 2022 Sep 6;10(9):1798. doi: 10.3390/microorganisms10091798 (PMC9501077; doi:10.3390/microorganisms10091798)

Table S1. Bacterial growth properties.

Strain	Colony colour	Colony morphology	*Anaerobic growth	pH range	Temperature range (°C)	PEG6000 (%) range
**RHF6** [1]	Creamy white	Flat	+++	4-10	15-50	0-15
**RHFS10** [2]	White	Undulate	++	6-12	15-50	0-15
**LS132**	Milky white	Translucent	++	2-10	25-40	0-15
**AGS172**	Creamy white	Wrinkled	++	2-10	25-50	0-20
**LMG9814**	Creamy white	Flat	++	4-10	25-60	0-15
**AGS84**	Creamy white	Flat	++	2-12	25-60	0-20
**AGS108**	Creamy white	Flat	++	2-12	25-60	0-20
**AGS54**	Creamy white	Irregular	+	4-10	4-40	0-15

*Anaerobic growth: +:low growth; ++:moderately growth; +++:high growth

Table S2. Summary of plant growth-promoting and biocontrol traits exhibited by the 8 bacterial strains.

	PGP traits						HYDROLYTIC ACTIVITIES (%)			
Strain	Swarming	PVK	IAA (μg/mL)	Ammonia production (mg/L)	Siderophores (%)	Biosurfactants	Protease	Amylase	Xylanase	CMC
RHF6[1]	+	++	4.5 ± 0.009	6.9 ± 0.03	7.1	+	100	100	41.7	100
RHFS10[2]	+++	++	6.5 ± 0.01	9.8 ± 0.02	41.7	+	100	100	76.9	100
LS132	+	-	1.4 ± 0.02	12.1 ± 0.1	16.7	-	100	0	0	0
AGS172	+++	+	12.9 ± 0.08	5.2 ± 0.001	11.8	+	100	100	25	100
LMG9814	-	+	8.6 ± 0.013	2.2 ± 0.02	4.5	-	100	75	41.2	37.9
AGS84	+	+	17.2 ± 0.21	4.1 ± 0.015	7.1	-	100	20	100	100
AGS108	++	++	5.7 ± 0.03	2.5 ± 0.1	3.1	+	100	64.3	22.2	33.3
AGS54	-	++	24.1 ± 0.3	2.7 ± 0.002	47.1	+	100	4	0	47.8

No activity (−), halo or colony diameter < 5 mm (+), halo or colony diameter 10 mm (+++). Data are represented by means of at least three replicates ± SE at $p \leq 0.05$ using LDS. PVK, Pikovskaya; IAA, indoleacetic acid; and CMC, carboxymethylcellulose. [1] Available from Petrillo et al. (2021). [2] Available from Castaldi et al. (2021).

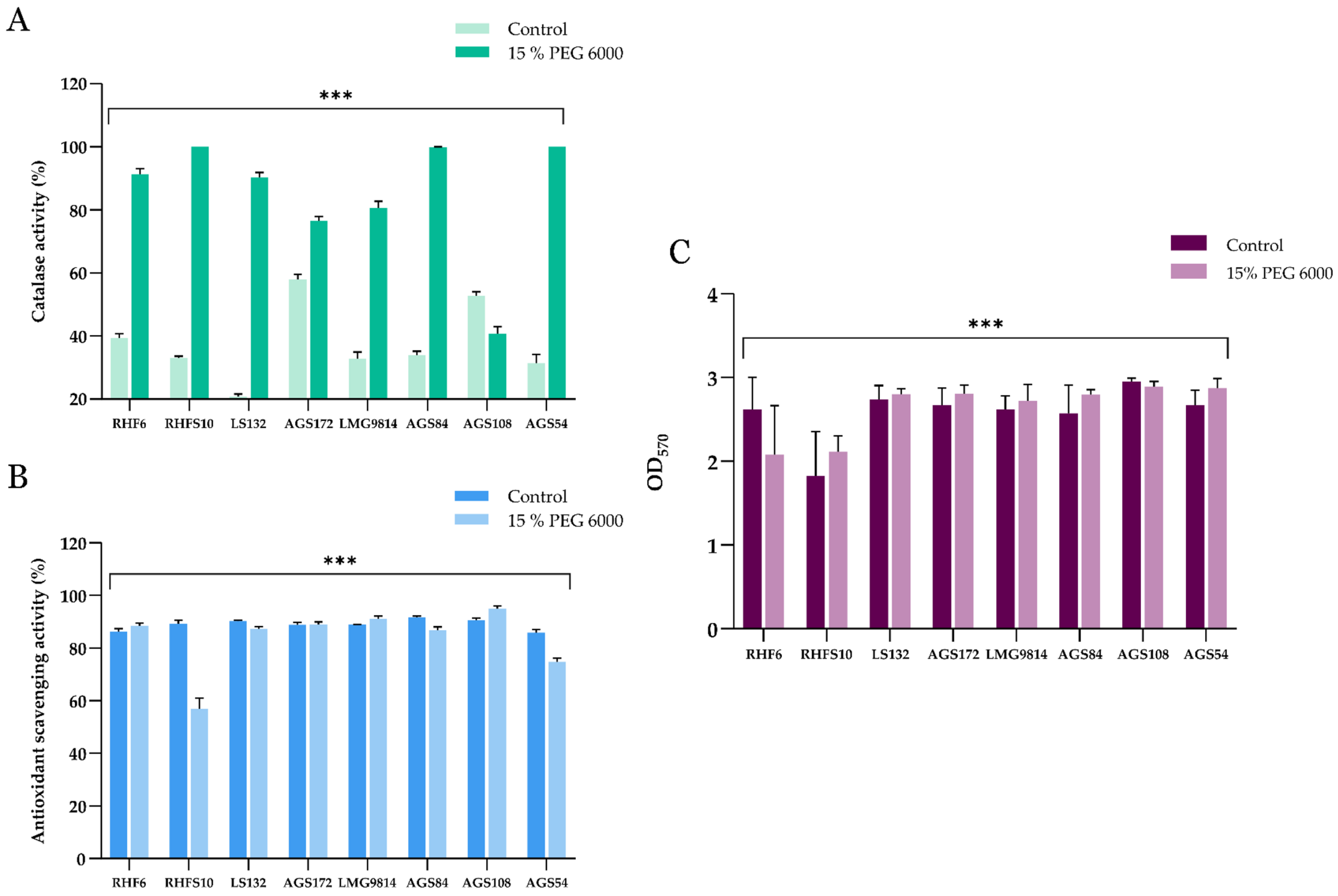

Figure S1. Bacterial antioxidant potential and biofilm production. Catalase activity (**a**), DPPH activity (**b**) and biofilm production shown as the absorbance of Crystal Violet registered at 570nm (**c**), assayed under either standard and drought stress (15% PEG 6000) conditions.

Table S3. *In vitro* bacterial compatibility.

Strain	RHF6	RHFS10	LS132	AGS172	LMG9814	AGS84	AGS108	AGS54
RHF6								
RHFS10	+							
LS132	-	+						
AGS172	+	+	+					
LMG9814	+	+	+	+				
AGS84	+	+	nc	-	+			
AGS108	+	+	+	+	+	-		
AGS54	-	+	+	nc	+	+	+	

+: compatible; -: incompatible; nc: not clear

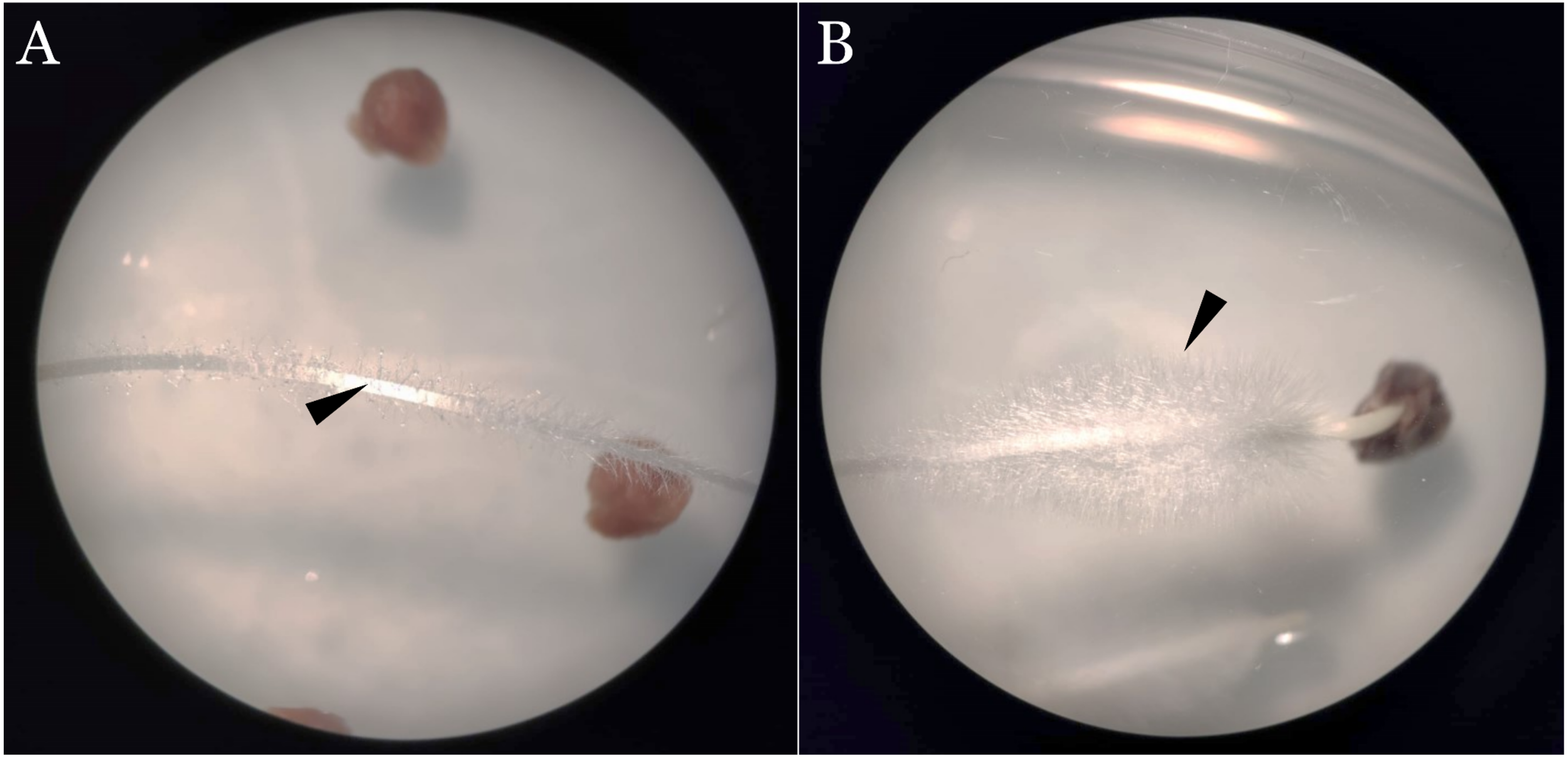

Figure S2. Representative pictures of lateral radicles of seedlings' roots, acquired by stereoscopic microscope (10X magnification) during the germination assay. (**a**) Seedlings treated with 1X PBS (control); (**b**) Seedlings treated with C2. Black arrows point at lateral radicles.

Figure S3. Comparison of the length of representative *S. oleracea* shoots treated with 1X PBS (C-) or the bacterial consortium (C2) after 27 days of growth under standard and drought conditions.

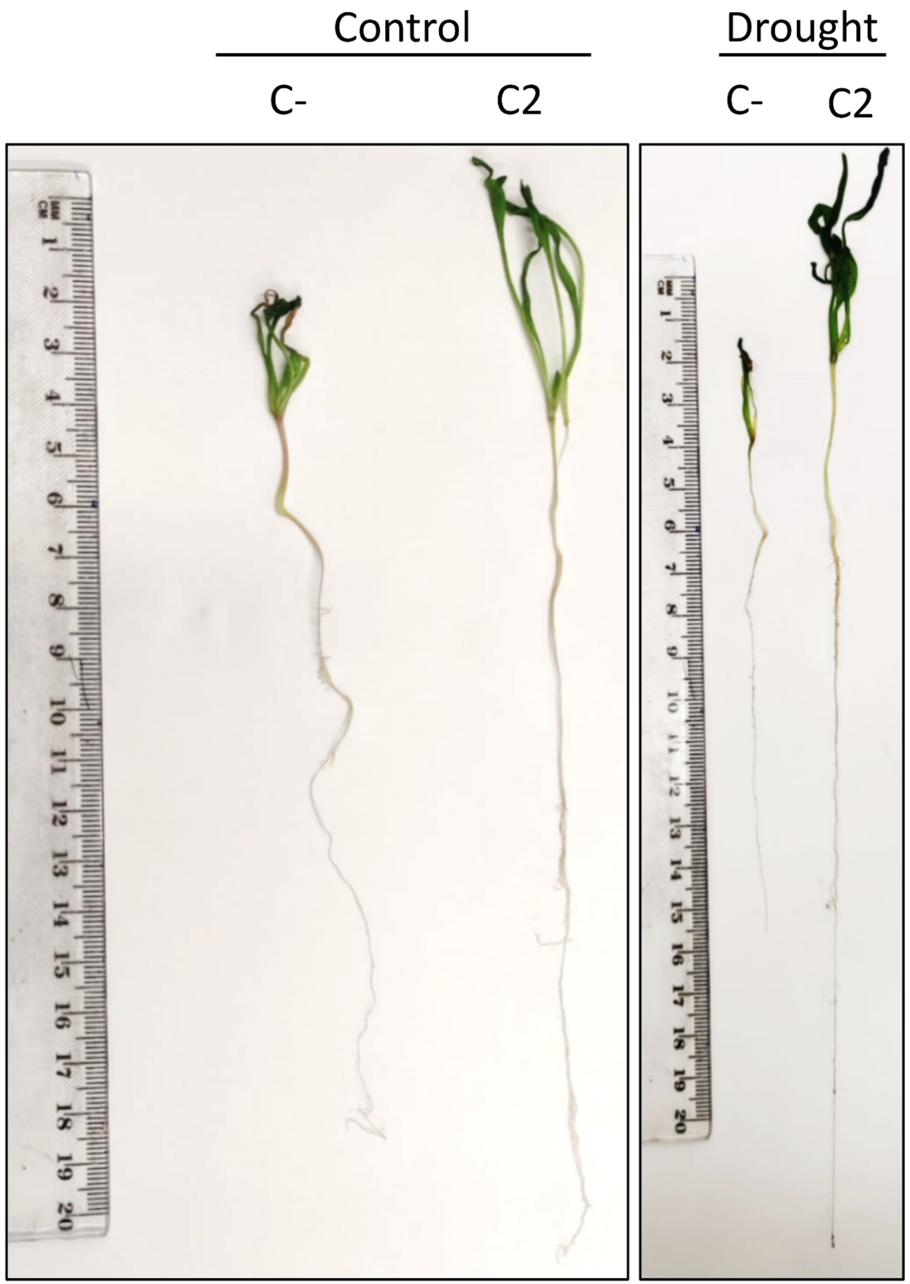

Supplement: Supplementary file 1 [file microorganisms-10-01798-s001.zip › microorganisms-1873199-supplementary.pdf]
